# Biofilm thickness controls the relative importance of stochastic and deterministic processes in microbial community assembly in moving bed biofilm reactors

**DOI:** 10.1098/rsfs.2022.0069

**Published:** 2023-02-10

**Authors:** S. Jane Fowler, Elena Torresi, Arnaud Dechesne, Barth F. Smets

**Affiliations:** ^1^ Department of Biological Sciences, Simon Fraser University, BC V5A 1S6, Canada; ^2^ Veolia Water Technologies, Lund, Sweden; ^3^ Department of Environmental Engineering, Technical University of Denmark, 2800 Kgs Lyngby, Denmark

**Keywords:** biofilm, community assembly, moving bed biofilm reactor, neutral model, nitrification

## Abstract

Deterministic and stochastic processes are believed to play a combined role in microbial community assembly, though little is known about the factors determining their relative importance. We investigated the effect of biofilm thickness on community assembly in nitrifying moving bed biofilm reactors using biofilm carriers where maximum biofilm thickness is controlled. We examined the contribution of stochastic and deterministic processes to biofilm assembly in a steady state system using neutral community modelling and community diversity analysis with a null-modelling approach. Our results indicate that the formation of biofilms results in habitat filtration, causing selection for phylogenetically closely related community members, resulting in a substantial enrichment of *Nitrospira* spp. in the biofilm communities. Stochastic assembly processes were more prevalent in biofilms of 200 µm and thicker, while stronger selection in thinner (50 µm) biofilms could be driven by hydrodynamic and shear forces at the biofilm surface. Thicker biofilms exhibited greater phylogenetic beta-diversity, which may be driven by a variable selection regime caused by variation in environmental conditions between replicate carrier communities, or by drift combined with low migration rates resulting in stochastic historical contingency during community establishment. Our results indicate that assembly processes vary with biofilm thickness, contributing to our understanding of biofilm ecology and potentially paving the way towards strategies for microbial community management in biofilm systems.

## 1. Introduction

The assembly of microbial communities is driven by a combination of niche-based processes, in which environmental conditions and microbial interactions impose selection, and stochastic processes, including random birth and death events (i.e. drift), dispersal and diversification [1,2]. Both deterministic and stochastic processes can play important roles in community assembly, with their relative importance depending on the strength of selection and the rate of stochastic dispersal. A number of recent studies on engineered microbial environments have described a combined role for stochastic and deterministic processes in community assembly [3–9]. Developing an understanding of the relative importance and contributions of these processes to community assembly under various conditions or designs could inform strategies for engineering and managing microbial communities and to predict patterns of resistance and resilience in the face of disturbance. We posit that deterministically assembled communities will have very different responses to disturbance compared to stochastically assembled communities, so an understanding of the controls on community assembly can improve our ability to make predictions and manage communities.

The biofilm lifestyle is believed to be the most prevalent form of microbial life [10]. Biofilms have important ramifications in a range of fields including medical microbiology, industrial microbiology and environmental engineering. Despite the importance of the biofilm lifestyle, the ecological processes involved in the assembly of biofilms from planktonic communities remain unclear. Engineered systems provide ideal model systems for studying the assembly of complex microbial communities due to the ability to closely control environmental variables and monitor dispersal. Biofilm-based microbial biotechnologies for drinking water and wastewater treatment often achieve similar contaminant removal efficiencies compared to suspended systems, with a lower footprint and less sludge production [11]. The enhanced microbial retention that occurs in biofilms allows for the growth of slow-growing organisms, which may result in improved functionality [12]. In wastewater treatment systems, biofilm communities tend to be more biodiverse than suspended ones, which can result in increased functionality and stability [13–15]. Different studies examining community assembly processes in biofilms draw diverging conclusions. For example, the assembly of mature stream biofilms appears to be mainly deterministic [16,17], while the formation of biofilms on microbial electrolysis cells (MECs) in wastewater is dominated by stochastic processes [5,8,18]. The factors driving these diverging observations are not well understood, and many studies include additional selective pressures in addition to biofilm formation that can confound the identification of assembly processes involved solely in the process of biofilm development [3,6].

Biofilm thickness varies substantially in natural and engineered systems and is generally thought of as an emergent property, which is determined by hydrodynamic forces such as shear, substrate loading rates and the types of organisms present [19]. Although the role of biofilm thickness in biological treatment processes has been widely discussed and modelled [20–22], few studies have considered biofilm thickness as a parameter that can be controlled [23–27]. Controlling biofilm thickness could prove useful for managing microbial communities in engineered environments. We therefore sought to examine the relative importance of deterministic and stochastic processes in the assembly of biofilm communities, and to investigate if controlling biofilm thickness influences community assembly processes. Biofilm formation can induce competition for space, which is likely strongest in the thinnest biofilms. With increasing biofilm thickness, nutrient gradients become larger, creating contrasting microenvironments which can support a greater diversity of metabolic lifestyles, but may result in additional selective pressures on the organisms present. A previous study in which biofilms of two thicknesses were compared emphasized the importance of deterministic processes in driving the differences in biofilms of different thicknesses; however, stochastic processes were not explicitly considered [26]. We hypothesize that there is a substantial role for stochastic processes in biofilm assembly, and that while selection likely occurs in all biofilms, the type of selection may vary. We expect that resource and diffusion limitation will generate greater competition in biofilms than in planktonic communities. Intense competition for space and a homogeneous environment in thin biofilms could result in competitive exclusion, while increased variable selection may be observed in thicker biofilms due to environmental heterogeneity [28].

We examined established biofilms of five thicknesses ranging from 50 to 500 µm to assess differences in assembly processes between biofilms across a range of diffusion limitation, resource gradients and spatial constraints. We used Sloan's neutral community model to assess the extent of stochastic processes in biofilm assembly from the suspended metacommunity [29]. We then assessed the extent and type of deterministic processes acting on the communities using a null-modelling approach and identified the organisms driving these effects. Lastly, we considered the calculation of migration rates using the neutral model and considered the implications of migration rates on the importance of dispersal in microbial community assembly.

## 2. Methods

Biofilm thickness was controlled using AnoxK™ Z-carriers, saddle-shaped biofilm carriers with a grid-covered surface that dictates the maximum biofilm thickness. Constant abrasion by carriers within the reactors ensures that biofilms do not exceed the grid height [25]. We used carriers with five different grid heights: 50, 200, 300, 400 and 500 µm (corresponding to Z50 to Z500). Two continuous-flow nitrifying moving bed biofilm reactors (MBBRs) were run for approximately 350 days fed with unfiltered effluent from a wastewater treatment plant (Källby, Sweden; effluent data in the electronic supplementary material, table S1) supplemented with 50 mg l^−1^ NH_4_-N and 0.5 mg l^−1^ PO_4_-P as both substrate and biomass source. Reactor 1 (3 l) contained 200 carriers each of Z200, Z300, Z400 and Z500 carriers, and reactor 2 (1.5 l) contained 293 Z50 carriers. The reactors were run at the same nominal loading rates per carrier surface area (2 g N m^−2^ d^−1^). The actual average loading rates over the sampling period were 1.84 g m^−2^ d^−1^ and 2.23 g m^−2^ d^−1^ with average nitrogen removal rates of 88% and 92% for reactors 1 and 2, respectively. Temperature (20°C), pH (7.5), DO (4.5 mg l^−1^) and HRT (hydraulic retention time, 2 h) were the same for both reactors. This short HRT was selected to minimize homogenizing dispersal between carriers. The details associated with the operation of these reactors have been previously published in [27].

### 2.1. DNA extraction, qPCR and amplicon sequencing

Duplicates of carrier samples were taken once ammonium removal was stable at T1 (day 168 R1 and day 123 R2) and T2 (day 275 R1 and 230 R2) for all carriers, and a single carrier was taken at T3 (day 386 R1 and 341 R2) for Z50, Z200, Z400 and Z500. The influent to each reactor was sampled five times, from two different batches (R1 days 271, 273, 276, 278, 284; R2 days 226, 228, 231, 233, 239). As previously described [27], biomass was detached and collected using a sterile brush (biofilm) or centrifuged (suspended) and was subjected to DNA extraction with the MP Biomedicals FastDNA Spin Kit. Total cell numbers in each sample were quantified by qPCR of the 16S rRNA gene using primers 1055F and 1392R [30,31]. Briefly, 10 ng of DNA, 12.5 µl of 2× iQ SYBR Green Supermix (Bio-Rad), and 500 nM of each primer were added to DNA-/RNA-free water in a total reaction volume of 25 µl. Thermocycling conditions were as previously described [32]. Total copy numbers were converted to cell numbers using CaRcone (https://github.com/ardagulay/CaRcone—Community-average-rRNA-gene-copy-nr-estimator) to predict 16S rRNA gene copy number from taxonomic classification of 16S rRNA gene amplicon sequencing data using rrnDB [33]. This method determines an average copy number per sample by assigning the average copy numbers from rrnDB to sequence variants based on taxonomic classification matches between rrnDB and the samples. For amplicon sequencing, the V3-V4 region of the 16S rRNA gene was amplified using PRK341F and PRK806R [34]. PCR purification with AMPure XP beads (Beckman-Coulter), library preparation and sequencing by MiSeq Illumina were performed at the DTU Multi-Assay Core Centre (Kgs Lyngby, Denmark).

### 2.2. Bioinformatics and statistical analysis

Quality control, merging of paired ends and error correction were performed in DADA2 (v1.6) [35]. Amplicon sequence variants (ASVs) were classified with SILVA v128. Samples were rarefied to 37 378 sequences, the number of reads in the sample with the fewest reads, for further analysis. Downstream data analysis and visualization were performed in R using phyloseq (v1.24), phangorn (v2.4), vegan (v2.5-1), Hmisc (v4.1-1), picante (v1.6-2), MicEco, ade4 (v1.7-11), adegraphics (v1.0-12) and ggplot2 packages [36–40]. Neutral community modelling was used to assess the extent to which community structure could be explained by stochastic assembly processes [29,41]. The source community consisted of the influent to the reactors, and the target community was the relevant carrier communities. The neutral community model on all biofilms was run ten times with a total of five carrier samples in each run so that results would be comparable to the models run for each carrier group (with a carrier of each thickness in each run). The mean of the ten runs was then taken (table 1; electronic supplementary material, table S2). The neutral model was then applied to carriers of each thickness. To characterize deterministic processes, phylogenetic alpha diversity (Faith's phylogenetic diversity (PD)) and beta-diversity (beta-mean nearest taxon distance (*β*MNTD) and beta-mean pairwise distance (*β*MPD)) were compared to null models (999 runs) made up of the whole ASV pool being considered using a maximum-likelihood tree generated and optimized in phangorn. Thus, the values reported here are *β*NTI (beta nearest taxon index) and *β*NRI (beta net relatedness index), which describe the phylogenetic structure of communities focusing on the tips of the trees and overall tree, respectively, relative to the null distribution [42]. Abundance-weighted *β*NTI and *β*NRI were measured using the ses.comdistnt and ses.comdist functions in the MicEco package (v. 0.9.11). These functions implement the comdist and ses.mpd functions and the comdistnt and ses.mntd functions from picante, but the package allows for multicore processing. We used the richness null model, which maintains sample richness by randomizing the ASV abundance matrix within samples. We previously found richness to increase with biofilm thickness [27] and thus found it to be important to maintain this parameter in the null model. The selection of null model can have a large effect on the outcome of these measurements. The richness null model has been shown to have high sensitivity for detecting habitat filtering and competitive exclusion, but can be prone to type I errors [43]. Thus we are quite confident that any observations of neutrality detected using this null model are quite robust. For all phylogenetic metrics compared to a null model, a negative value denotes a greater degree of clustering than expected by chance, and a positive value denotes overdispersion. Differences from the null distribution are considered significant when values are greater than 2 s.d. from the mean. Double principal coordinate analysis (DPCoA) was conducted in Phyloseq using the dpcoa function. Weighted Unifrac analysis was performed in Phyloseq using the ordinate function. Sequence data from this study have been deposited to Genbank under PRJNA322602.

**Table 1.** Results of neutral modelling.

	all	Z50	Z200	Z300	Z400	Z500
model fit (Spearman *ρ*)	0.495	0.298	0.561	0.546	0.476	0.456
ASVs neutral (modelled)	746.6 (1746)	391 (526)	810 (922)	955 (1020)	762 (881)	698 (815)
relative abundance (%) of neutral ASVs	N/A	41.5	74.9	74.1	69.9	69.8
number of ASVs selected for	62.7	87	48	36	55	62
relative abundance (%) of ASVs selected for	N/A	22.7	2.4	1.4	3.9	5.5

## 3. Results

### 3.1. Thicker biofilms exhibit greater stochasticity in assembly processes

**Figure 1.**
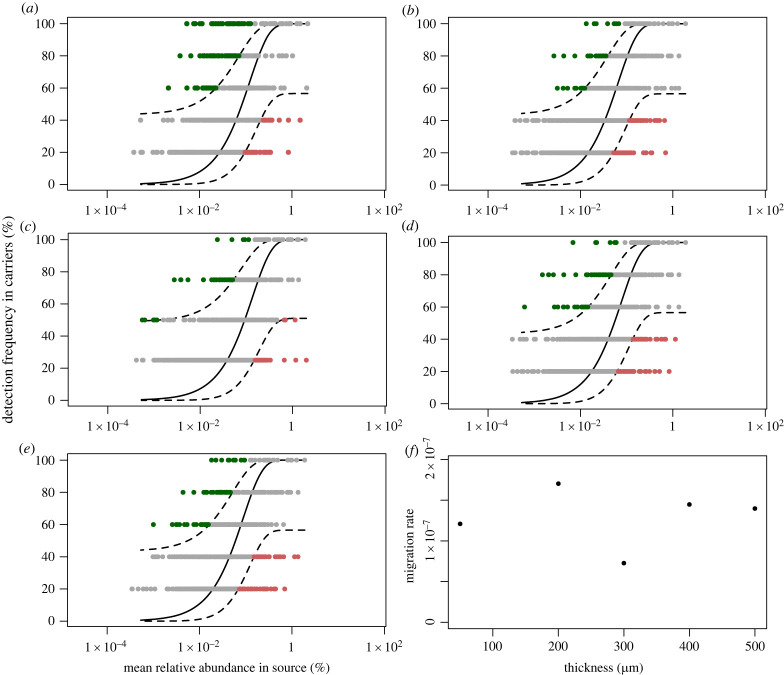
Neutral model output for Z50 (*a*), Z200 (*b*), Z300 (*c*), Z400 (*d*) and Z500 (*e*). The solid line shows the model fit, and the dotted lines represent 95% confidence intervals. Neutrally assembled ASVs are shown within the confidence intervals in grey while those that are selected for or against in the carriers relative to the influent are in red and green, respectively. (*f*) The relationship between biofilm thickness and the migration rate normalized by surface area. Migration rate was calculated based on the *N*_T_*m* parameter from the neutral model output where *N*_T_ (community size) was measured by qPCR of total bacteria 16S rRNA genes of carrier groups.

The extent to which biofilm community structures can be explained by stochastic processes was investigated by applying the neutral community model [29]. When considering all of the biofilm carrier communities compared to the incoming planktonic community, the community compositions fit the neutral model well. However, when considering biofilms by thickness, those that were 200 µm and thicker had a better fit than the 50 µm biofilms (table 1, figure 1). The poorer fit of the 50 µm biofilms with the neutral model is due to the large number of ASVs (87) that were selected for (in green in figure 1*a*), which make up 23% of the 50 µm biofilm communities. In thicker biofilms, between 86 and 94% of ASVs, comprising 70–75% of the communities in relative abundance, fit the neutral model while for 50 µm biofilms, only 74% of ASVs, comprising 42% of the relative abundance, did. The fit of the neutral model is determined by the *N*_T_*m* parameter, which is the product of the local community size (*N*_T_) and the migration rate (*m*). Using qPCR of the 16S rRNA gene for total bacteria to determine total cell numbers in the biofilms (electronic supplementary material, table S3), we were able to calculate the migration rate for each thickness [44]. The migration rate is the probability that an unfilled space in the community is filled by a microbe from the source community rather than by cell division within the community. Overall migration rates were low, ranging from 7.36 × 10^−8^ to 1.71 × 10^−7^, suggesting that the establishment of cells from the influent community to the biofilms was infrequent. Migration rates did not vary substantially with biofilm thickness (figure 1*f*).

### 3.2. Habitat filtering plays a role in all biofilms

**Figure 2.**
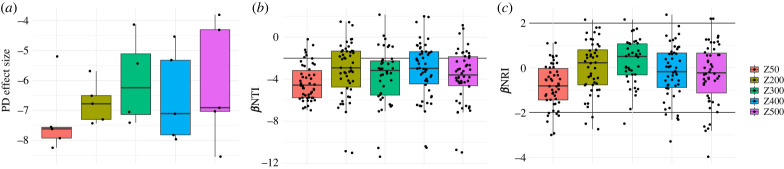
Phylogenetic diversity of carrier communities Faith's PD (*a*), *β*NTI (*b*) and *β*NRI (*c*) relative to influent communities. Results are presented as the standardized effect size relative to a null model composed of the source (influent) community. Negative values reflect phylogenetic clustering relative to the influent, and positive values reflect overdispersion. Values greater than |2| are considered to be significantly different from the null model.

**Figure 3.**
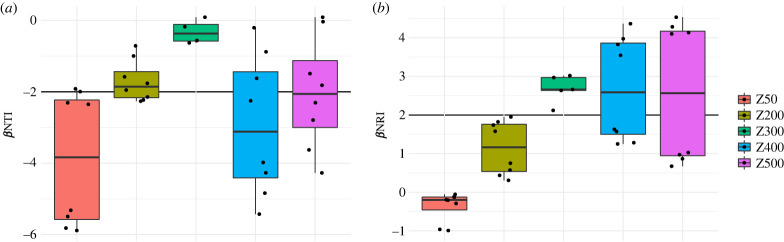
Within group phylogenetic diversity *β*NTI (*a*) and *β*NRI (*b*) of carrier communities. Results are presented as standardized effect size relative to a null model that consists of all the communities from a carrier group. Negative and positive values denote that communities are more or less similar to each other than expected by chance (i.e. homogeneous or variable selection).

The implemented neutral model only considers ASVs that are shared between the influent and the carrier communities. Thus, it does not provide a complete view of community assembly, as it disregards any ASVs detected in the influent but not in the carriers, and vice versa. We therefore examined the phylogenetic structure of the communities to assess the role of selection in the formation of the biofilms [42]. Standardized effect sizes of Faith's PD were calculated by comparing PD to a null model consisting of the influent community and relevant biofilm thickness samples (i.e. comparing to complete source and target communities). The results show that all of the biofilm communities are more phylogenetically clustered than expected by chance, with average effect sizes ranging from −6.0 to −7.3. The greatest clustering was observed for the 50 µm biofilms, but this was not significantly different from other thicknesses (figure 2*a*). To further examine selection processes, we calculated phylogenetic beta-diversity metrics *β*NTI and *β*NRI between the influent communities (source) and the biofilms (target). The *β*NTI showed that in all biofilm types, community members were more phylogenetically clustered relative to the influent than would expected by chance, with the 50 µm biofilms again exhibiting the greatest degree of clustering (figure 2*b*). Comparing the overall phylogenetic breadth of communities of the influent and carrier communities (*β*NRI) revealed that the 50 µm biofilms were more clustered compared to the other thicknesses (ANOVA, Tukey, *p*_adj_ = 0.00026–0.62, Z200–Z400), but none of the communities exhibited clustering that was significantly different from the null model expectation (figure 2*c*). Together these results point towards habitat filtering across all biofilms, particularly at short phylogenetic distance, and with the greatest effects in the thinnest biofilms. Since biofilm carriers with the same maximum thickness could be considered as replicate communities, we measured *β*NTI and *β*NRI within each of the carrier groups to distinguish between homogeneous or variable selection and neutral scenarios [28]. The 50 µm biofilms were more similar to each other at short phylogenetic distances than expected by chance, but the broader phylogenetic structure was not substantially different from the metacommunity (figure 3). By contrast, thicker biofilms less frequently exhibited clustering at short phylogenetic distances, although in the thickest biofilms (400–500 µm), selection for closely related bacteria seemed to increase somewhat (figure 3*a*). On the other hand, biofilms thicker than 200 µm exhibited overdispersion with respect to the overall phylogenetic structure relative to the metacommunity (figure 3*b*).

**Figure 4.**
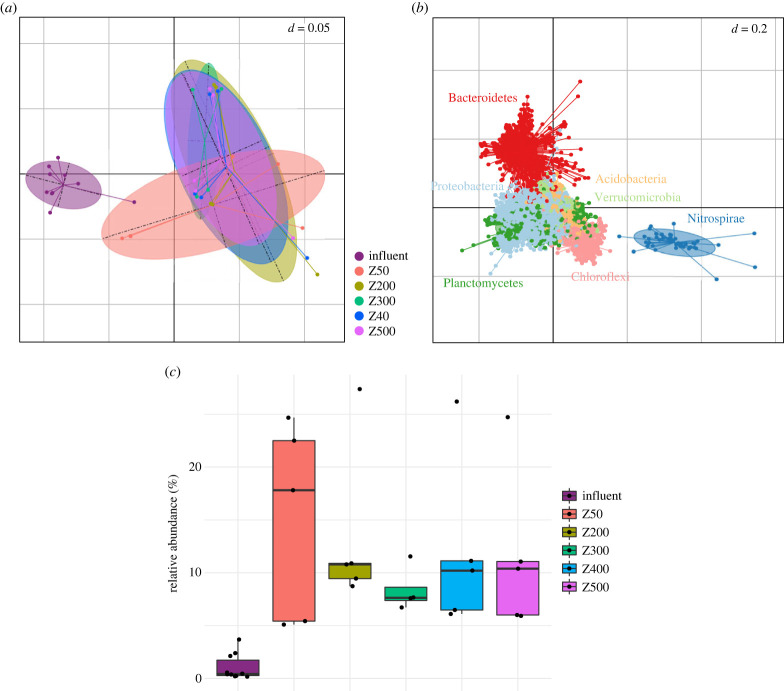
DPCoA of biofilm and influent communities (*a,b*). The enrichment of *Nitrospira* spp. in biofilm communities can be seen in (*b*,*c*). The horizontal and vertical axes explain 43% and 18% of the variation. (*c*) Relative abundance of *Nitrospira* spp. in carrier and influent communities.

These results point towards different assembly patterns between the thin and thicker biofilm communities. To identify the taxa that drove these differences, we performed a DPCoA (figure 4) [45]. Ordination by DPCoA shows a clear separation of the biofilm and non-biofilm communities. Differences between the biofilm and non-biofilm communities are driven by selection for members of the phylum Nitrospirae in the biofilm communities (figure 4), which are the main beneficiaries of the habitat filtering observed in all biofilms relative to the influent (figure 2*a,b*). It is worth noting that all members of phylum Nitrospirae detected belong to the genus *Nitrospira* and are canonical nitrite-oxidizers, as *amoA* genes of *Nitrospira* were not detected by qPCR (data not shown) [32]. We further performed a constrained analysis (cDPCoA) to try to identify the taxa that differentiate biofilms of different thicknesses. This analysis revealed clear differences between 50 µm and thicker biofilms that are driven by members of diverse phyla but failed to separate the thicker biofilms from each other (electronic supplementary material, figure S1).

## 4. Discussion

Our results point towards a combined role of stochastic and deterministic processes in the assembly of MBBR biofilm communities, with biofilm thickness driving their relative importance. Importantly, the assembly of a large proportion (approximately half) of the members of biofilms 200 µm and thicker can be explained by stochastic processes, accounting for up to 75% of biofilm relative abundance (table 1), indicating that stochastic processes play a major role in biofilm assembly. In addition, habitat filtering, resulting in, in particular, the enrichment of nitrite-oxidizing *Nitrospira* spp. relative to the influent was observed in all biofilms (figure 4). Environmental differences between maximum biofilm thicknesses appeared to impact the importance of and the type of deterministic processes involved in community assembly. Thin biofilms were more similar to each other than expected by chance suggesting a highly uniform environment in which phylogenetically similar organisms are selected for, reflecting a homogeneous selection regime [28]. Conversely, thick biofilms tended towards phylogenetic overdispersion which could be explained by either deterministic or stochastic processes. A variable selection regime due to variations in substrate gradients within and between replicate communities could explain this observation [28]. On the other hand, given the low migration rates and greater role of stochastic processes in thicker biofilms, stochastic attachment and low migration rates followed by drift could create historical contingency effects resulting in differences between replicate community compositions, as has been suggested for the developing rumen microbiome [46] and MECs [8]. Distinguishing between these two scenarios driven by either deterministic or stochastic processes is not possible, as both could cause the observed patterns.

The differences in community assembly processes in biofilms of different thicknesses also resulted in functional differences where thin biofilms had high nitrification rates and thicker biofilms were less efficient at nitrification, but showed improved transformation of diverse micropollutants [27]. Another study comparing both thin (50 µm) and thick (400 µm) biofilm communities in an MBBR for nitrogen removal found that the communities of different thicknesses were distinct and attributed this to deterministic processes [26]. While our results also show distinct biofilm communities with thickness, our results give a more nuanced view of assembly processes by considering the source community composition, explicitly considering neutral processes and examining biofilm thickness in greater resolution. Both studies agree that biofilm thickness can influence both community composition and function, and we suggest that this is related to different community assembly processes between thin and thick biofilms.

The reactors in this study were inoculated and operated with effluent wastewater. The addition of ammonia to the effluent generated a selective pressure for nitrifying bacteria and against heterotrophs, and this manifested in the enrichment of *Nitrospira* spp., and the maintenance of high numbers of *Nitrosomonas* spp. in all biofilms. However, other selection pressures were minimized. The wastewater community used was well adapted to the environment provided. The good agreement with the neutral community model suggests that many microbes are well adapted to the biofilm lifestyle, and that biofilm formation in itself is a relatively stochastic process. This is not particularly surprising if we consider that biofilms are the dominant lifestyle of microbes in nature [10]. Our observations are also in agreement with a previous study in which high levels of stochasticity were observed in the assembly of syntrophic fatty acid degrading biofilms when other selection pressures were weak [7]. Compared to studies that used the neutral community model to study assembly of biofilm communities, we find a better fit to the model than drinking water biofilter communities on granular activated carbon and sand [3] and glass beads [47] but poorer fits than MECs in wastewater [5]. The studies on drinking water, however, included additional selective regimes that likely contributed to increased importance of deterministic processes in community assembly. In particular, the low- and high-nitrite feeding regimes in [47] resulted in only slightly poorer fits with the neutral model (Spearman *ρ*, low nitrite = 0.39, high nitrite = 0.46) compared to the biofilms described here (*ρ* = 0.495). Given that different selective regimes were deliberately imposed by varying substrate concentration in that previous work, it is surprising that the thin biofilms described in our study exhibited a much poorer fit with the neutral model (*ρ* = 0.295). Thus, limiting biofilm thickness to this extent may impose considerable selective pressure. We propose that hydrodynamic forces and physical shearing at the biofilm surface, combined with competition for space, may provide an explanation for the stronger selection observed in thin biofilms. The surface area to volume ratio of the thin biofilm was between 4- and 10-fold higher than that of the thicker biofilms. Previous work has shown the importance of hydrodynamic forces as a selective parameter in microbial communities [48]. Based on previous modelling efforts, it is believed that 50 µm and thinner biofilms would not have substantial substrate gradients [26], and thus the environment would be homogeneous with depth. If hydrodynamic forces were strong at the surface of the biofilms, it is likely that only the fastest growing taxa that were capable of persisting despite hydrodynamic shear and abrasion, or alternatively were able to penetrate deeper biofilm layers, would persist in the thinnest biofilms.

### 4.1. Migration to the biofilms is low

The rate of migration of members from the source community to the biofilm can be determined using the neutral model if the size of the target community (*N*_T_) is known. We considered each carrier as a single microbial community and quantified community size using 16S rRNA gene-based qPCR. We used the method described by Sloan *et al*. [44] to calculate the true migration rate of a community when a subsample of the community (of size *N*_s_) is used to calibrate the model. As determined by this method, *m*, the probability that an empty space in the community will be filled by an organism from the source community, was low for all biofilms, ranging from 7.38 × 10^−8^ to 1.17 × 10^−7^ (figure 1*f*). Extremely low *m* values have also been noted by other studies that used realistic values of *N*_T_ [9]. However, few studies have calculated migration rates predicted by the neutral model, and those that have often ignore sampling effects, and use the collected sample size as *N*_T_, rather than the true size of the target community. When using the sample size (37 378 sequences after rarefaction) in order to compare to other studies, the migration rates in this study varied from 0.0033 to 0.0067. By comparison, migration rates estimated for other environments tend to be much higher, including the Humber estuary (0.7 for both AOB and denitrifiers), the respiratory tract (0.2) and a sewage treatment plant (0.1) [29]. Migration rates in the human gut were somewhat lower, but still an order of magnitude greater than in this study (0.032–0.063) [49]. However, these differences in reported migration rates appear to be inversely related to the sample size with samples as small as 13 clones [29]. Indeed, in another study of neutral assembly of microbial communities in tree holes, *N*_T_ was estimated based on tree hole volume and microbial density, and the migration rate was several orders of magnitude lower, estimated at 1 × 10^−6^ [50]. Sloan *et al*. [44] recalculated *m* for several environments taking into account the sampling effect. Corrected *m* values for the Humber estuary and sewage samples are 7.0 × 10^−8^ and 1.55 × 10^−9^, respectively, given an *N*_T_ of 10^9^ cells, even lower than for the biofilm communities in the present study. The migration rates in all of these studies seem low intuitively, but since migration rates in microbial communities have not been measured experimentally, it is not possible to comment on whether this is an accurate estimate. Accurate estimations of *N*_T_ and *m* could enhance our ability to understand community assembly processes leading to predictive microbial community management. The effective community size (*N*_ec_) is a relatively new concept that could be used to reflect ecologically relevant microbial community sizes to use as *N*_T_ [51]. This method may also provide more realistic estimates of *m* going forward.

Mature biofilms on carriers with a maximum biofilm thickness would be difficult to colonize once established, as migrating cells would quickly be sloughed off due to hydrodynamic and physical forces acting at the biofilm surface. Had we sampled at earlier timepoints during biofilm establishment, before steady state, we may have observed higher migration rates when these forces were weaker. Supporting this idea, a study examining biofilm succession from initial establishment found that bacterial community assembly was driven primarily by stochastic processes, but that a high level of replacement occurred as biofilms developed [52]. In the mature biofilms that we sampled, we never observed a pattern between maximum thickness and migration rate. This indicates that biofilm thickness did not influence the migration rate, and we suggest that surface area is likely to be a more important control on migration in biofilms experiencing high shear forces.

### 4.2. Steady state communities were not observed

The high variability in community structure between biofilms of the same thickness reveals that substantial changes in community structure occurred over time (figure 3; electronic supplementary material, figure S2) even though biofilms were only sampled once nitrogen removal had reached steady state [27]. Since the community had reached a functional steady state, we expected that the community composition would be similarly consistent; however, this was not the case. Replicate communities were always similar to each other, but composition varied over time. This is not believed to be due to variation in the influent community (wastewater treatment plant effluent) as the influent microbial community composition was observed to be highly consistent even between collected batches (electronic supplementary material, figure S2). Given the low migration rates, it is not expected that the influent composition would have had a major impact on biofilm composition once they were established. Thus, the observed variations in biofilm composition are likely due to drift or selection. Variation of biofilm communities over time is predicted by models of biofilm succession based on both neutral and niche perspectives [53,54]. However, these models focus mainly on the initial establishment of biofilms and not on changes occurring after the initial colonization and development stages. One study that examined the establishment of MBRs over 30 days also observed temporal variation in communities and in assembly processes; however, given the short timeframe of that study, early biofilm succession may still have been occurring in the communities [6]. Further work examining long-term biofilm succession is needed to define the relative importance of drift, migration and various deterministic processes over time.

A drawback of the experimental design of this study is that the thinnest biofilms (50 µm) were in a different reactor than the thicker biofilms, started 45 days later. It is unlikely that the observed differences can be explained entirely by the existence of two reactors as general trends in reactor performance showed consistently increasing nitrification rate and decreasing micropollutant removal with thickness. The feed and thus metacommunity entering the reactors were identical (and were stable over time), as were the hydraulic-loading characteristics. The experimental design of the reactors was such that the HRT was short (2 h), thereby minimizing homogenizing dispersal between carriers. Additional insights could be gained by repeating this experiment with all carrier thicknesses in a single reactor, and all carrier thicknesses in individual reactors.

## 5. Conclusion

The assembly of MBBR biofilms from planktonic communities involves a combination of stochastic and deterministic processes that vary in their relative importance with thickness. The role of dispersal in community assembly of mature biofilms appears to be small. The migration rate derived from the neutral model suggests that dispersal played a minor role in assembly, and that it did not vary between biofilm thicknesses. We suggest that in established biofilms subjected to shear forces, surface area is likely a more relevant factor than thickness in determining migration rates. Thus, drift is the main stochastic process driving the assembly of biofilms, and it can explain a large proportion of the community composition, particularly for biofilms of 200 µm and thicker. In addition to drift, selection also played a role in biofilm assembly. All biofilms exhibited habitat filtering compared to the suspended source community, with greater prevalence in thinner biofilms. Phylogenetic analysis points towards different selective pressures acting on biofilms of different thicknesses. In thin biofilms, homogeneous conditions throughout the biofilms and between replicates resulted in uniform communities in which closely related community members were selected for. For thicker biofilms, dissimilar replicate communities in which diverse members were present may have been due to variable selection or drift. Functionally, this resulted in thin biofilms with extremely efficient nitrification and thicker biofilms with less efficient nitrification but improved micropollutant degradation [27].

Better efforts to test neutral community models using well-defined source communities will reveal the conditions under which neutral processes play an important role [29,54], and similarly, using defined source communities in null modelling can provide insights into the strength and type of selective processes acting on communities. Few studies to date have used a defined source community to test the neutral community model, and even fewer have estimated migration rates. The quantification of migration rates is further complicated by the challenge of defining local community sizes and/or effective community sizes. A deeper consideration of what constitutes a microbial community and measurement of migration rates in different types of environments is needed. Further work on biofilm community assembly should aim to describe community assembly processes over time to unify neutral and niche-based models of biofilm succession [53,54] and to determine if steady state biofilm communities exist, or if they are in a constant state of flux.

## Data Availability

Sequence data are available in GenBank under PRJNA322602. Standard code for the reported R packages was used; any modifications are indicated in the Methods section. The data are provided in the electronic supplementary material [55].
